# Hereditary Hyperferritinemia-Cataract Syndrome in a Family With HFE-H63D Mutation

**DOI:** 10.7759/cureus.36253

**Published:** 2023-03-16

**Authors:** Tansu Eris, Ahmet Mert Yanik, Derya Demirtas, Asu Fergun Yilmaz, Tayfur Toptas

**Affiliations:** 1 Hematology, Marmara University School of Medicine, Istanbul, TUR; 2 Hematology, Marmara University Pendik Training and Research Hospital, Istanbul, TUR

**Keywords:** ferritin, cataract, hereditary hemochromatosis, hyperferritinemia, hereditary hyperferritinemia-cataract syndrome

## Abstract

Hereditary hyperferritinemia-cataract syndrome (HHCS) is a rare genetic condition characterized by persistent hyperferritinemia (usually ferritin >1,000 ng/mL) without tissue iron overload, with or without early-onset slow-progressing bilateral nuclear cataract. It was first identified as a new genetic disorder in 1995, and since then genetic sequencing studies have been carried out to identify associated mutations in affected families. New mutations around the world are still being reported in the iron-responsive element (IRE) of the L-ferritin gene (*FTL*) to this day. Many clinicians remain unaware of this rare condition. The co-occurrence of *FTL* mutations and hereditary hemochromatosis (HH) mutations, especially H63D, on the *HFE* gene has been reported in the literature, which often leads to a diagnosis of HH, missed diagnosis of HHCS, incorrect treatment with phlebotomies and the occurrence of associated iatrogenic iron deficiency anemia. We herein report the case of a 40-year-old woman with spontaneous facial freckling, bilateral cataracts, homozygosity for *HFE* H63D mutation, iron deficiency anemia, and hyperferritinemia, who has been treated with phlebotomy and iron chelation therapy to no avail. Eleven years after being diagnosed and treated for HH, a reevaluation of her clinical presentation, laboratory results, medical imaging, and family history led to the recognition that her case is explained not by HH, but by an alternative diagnosis, HHCS. Our main objective in this report is to increase clinical awareness about HHCS, an often-unknown differential diagnosis of hyperferritinemia without iron overload, and to prevent adverse medical interventions in HHCS patients.

## Introduction

Hereditary hyperferritinemia cataract syndrome (HHCS) is an extremely rare genetic disorder with autosomal dominant inheritance, and an estimated prevalence of 1/200,000 [1]. It was first described in 1995 separately by Bonneau and Girelli as a new genetic disorder that involves the ferritin light chain (L-ferritin) *FTL* gene on chromosome 19q, in the vicinity of the gene that codes for the lens membrane protein [2,3]. HHCS is characterized by hyperferritinemia without iron overload, with or without slow-progressing early-onset bilateral nuclear cataract. There are around 100 cases reported worldwide from many countries and continents. Here, we report on a new case of HHCS, whose initial presentation was atypical with spontaneous facial freckling, bilateral nuclear cataracts, hyperferritinemia without iron overload, and homozygosity for hereditary hemochromatosis (HH) *HFE* H63D mutation. Her symptoms were attributed to HH and treated as such before her clinical presentation, laboratory results, medical imaging, and family history were reassessed, and a new diagnosis of HHCS was reached 11 years after her first presentation.

## Case presentation

A 40-year-old woman was diagnosed with slow-progressing early-onset bilateral cataracts and HH with homozygosity for *HFE* H63D mutation confirmed with genetic testing in 2011. Despite being treated with regular phlebotomies from 2011 to 2013, and then again with iron chelation therapy in 2020, her hyperferritinemia and iron deficiency anemia persisted. The patient presented to different centers and was lost to follow-up several times over the course of 11 years. She presented to our hematology outpatient clinic in May 2022 to be evaluated for the need for phlebotomy. Her active complaints at this visit were deteriorating eyesight and persistent lethargy. Laboratory results at the last follow-up visit from one day prior (May 2022) and one month prior (April 2022) are shown in Table 1.

**Table 1:** Laboratory results of the index case at the last follow-up and laboratory results from one month prior. Abbreviations: TIBC: Total iron binding capacity; Hgb: Hemoglobin; MCV: Mean corpuscular volume; AST: Aspartate aminotransferase; ALT: Alanine aminotransferase

April 2022	May 2022
Serum iron: 16 μg/L (reference: 37-145 μg/L); TIBC: 507 μg/L (reference: 228-428 μg/L)	Ferritin: 1,399 μg/L (reference: 7-282 μg/L); Hgb: 8.3 g/dL (reference: 12.0-17.0 g/dL); MCV: 61.5 fL (reference: 82.0-97.0 fL); AST: 18 U/L (reference: 10-37 U/L); ALT: 14.3 U/L (reference: 10-40 U/L)

Her current state with hyperferritinemia, high total iron binding capacity (TIBC), and microcytic anemia were unusual for a case of HH. The patient’s history of bilateral cataracts, the discrepancy between her laboratory results and what is expected in HH, and her ongoing hyperferritinemia without iron overload made us consider the alternative diagnosis of HHCS and prompted us to reevaluate the patient's medical records and family history retrospectively.

The patient’s first presentation was in 2011 to a dermatology outpatient at another tertiary care center because of spontaneous facial freckling. Laboratory workup at the time revealed ferritin of 964.1 μg/L and hemoglobin of 14.1 g/dL. She was referred to the internal medicine outpatient clinic at the same center for further investigation of hyperferritinemia. Screenings for potential causes of hyperferritinemia without iron overload - viral hepatitis, HIV, hemoglobinopathies - were undertaken, but none of the tests were revealing. The patient never used alcohol in her life and her BMI was 24 kg/m^2^ (normal: 18.5-24.9 kg/m^2^). She was then referred for genetic testing on suspicion of having HH and found to be homozygous for histidine-to-aspartic acid substitution at amino acid position 63 (H63D) in the *HFE* gene; no cysteine-to-tyrosine substitution at amino acid position 282 (C282Y) was found [4]. Subsequently the diagnosis of HH was made. Phlebotomy was initiated with anticipation of a reasonable response in the reduction of iron overload as monitored by serum ferritin concentration on an as-needed basis. She also presented to the ophthalmology clinic around the same time for deteriorating eyesight, and a diagnosis of bilateral cataracts was made. Before she was lost to follow-up in 2013, her last laboratory result revealed hyperferritinemia without iron overload, with a ferritin of 1,077 μg/L, hemoglobin of 10 g/dL, serum iron of 22 μg/L, and TIBC of 428 μg/L, and normal complete blood count with differential otherwise despite regular phlebotomies from 2011 to 2013. 

**Figure 1:**
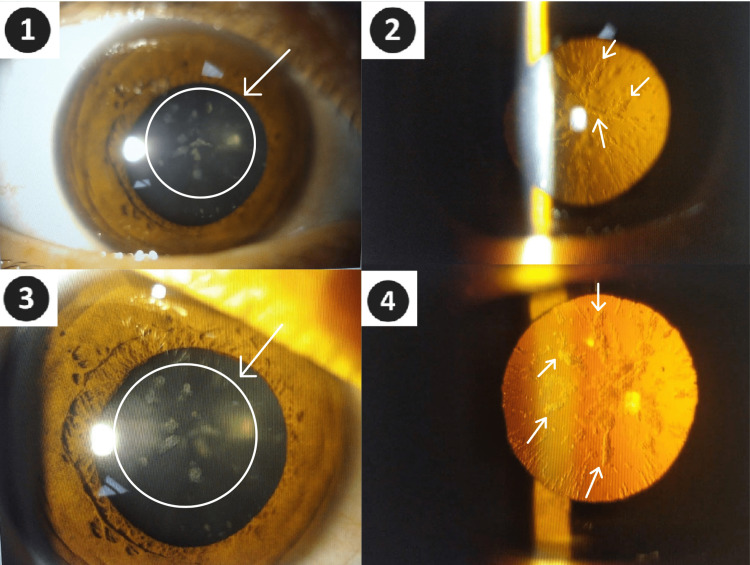
Slit lamp direct (1, 3) and retro-illumination (2, 4) displaying central and peripheral crystalline flecks in a radial pattern.

Per the patient’s medical records, she returned for continuing care at another center in July 2019. Her laboratory results revealed serum iron of 15 μg/L, and TIBC of 409 μg/L. In August 2020, the patient returned once again for care, and she was started on deferoxamine therapy based on her ferritin of 2,678 μg/L at the time and her noncompliance with phlebotomy. Six days later, on August 24, 2020, the patient had a sudden onset stomach ache, was unable to speak, vomited, and lost consciousness with a post-ictal phase. She was taken to the emergency department by her spouse, was intubated, and transferred to a tertiary care hospital with a presumed diagnosis of status epilepticus. After the patient was stabilized, it was thought that she might have developed a seizure due to the neurological involvement of HH, and was evaluated with an electroencephalogram, whole-body imaging, and laboratory workup. None of these tests were able to elucidate an etiology for her symptoms. She was also evaluated with a non-contrast-enhanced hepatobiliary MRI at that time, which demonstrated no evidence of iron overload. Before the patient was discharged, her last laboratory results revealed ferritin > 2,000 μg/L and hemoglobin of 9.6 g/dL, she was told to discontinue deferoxamine and called for follow-up in the internal medicine and neurology outpatient clinics in a month. She was lost to follow up again for two years and returned again in April and May 2022, which was the time we encountered the patient during her hematology outpatient visit. The most recent ophthalmologic evaluation of the patient with slit-lamp direct and retro-illumination revealed cataracts with a unique morphology, displaying central and peripheral crystalline flecks in a radial pattern (Figure 1), which was consistent with the HHCS cataract morphology as described in the literature [1].

Furthermore, we investigated the patient’s family history. She was one of five children born out of a non-consanguineous marriage in a village in the Central Anatolian region of Turkey. The patient had three sons currently aged 20, 18, and nine years. After the patient was diagnosed with HH, her three children underwent genetic testing. The eldest two sons were heterozygous for H63D, and the youngest son’s test result was never obtained by the family. All three children had laboratory-confirmed hyperferritinemia without iron overload at their initial testing (Table 2).

**Table 2:** All three children of the index case had laboratory-confirmed hyperferritinemia without iron overload at their initial testing. Each member of the family is identified by 2 numbers based on the pedigree in Figure 1. Roman numerals indicate generation and Arabic numerals indicate individuals in each generation listed by age. Abbreviations: M: Male, CRP: C-reactive protein, TIBC: Total iron binding capacity

Family member	Sex	Age at the time of laboratory testing	Ferritin level (μg/L)	Additional relevant laboratory findings
III.6	M	11	1,724	Hemoglobin: 13.6 g/dL (reference: 11-13.3 g/dL); Serum iron: 112 μg/dL (reference: 65-175 μg/dL); TIBC: 349 μg/dL (reference: 141-514 μg/dL)
III.7	M	13	1,962	Hemoglobin: 15 g/dL (reference: 12-16 g/dL); Serum iron: 113 μg/dL (reference: 65-175 μg/dL); TIBC: 431 μg/dL (reference: 120-530 μg/dL)
III.8	M	3	1,209.7	Hemoglobin: 11.6 g/dL (reference: 13.5-18.0 g/dL); Serum iron: 112 μg/dL (reference: 65-175 μg/dL); TIBC: not available; CRP < 3.3 mg/L (reference: 0-5 mg/L)

**Figure 2:**
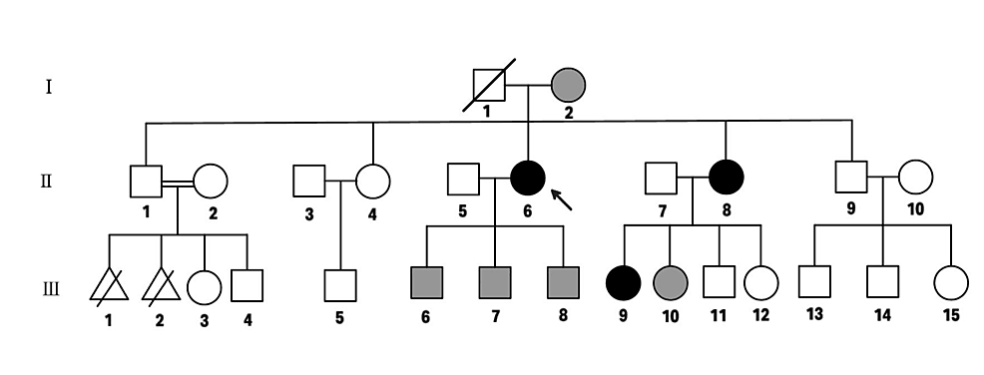
Pedigree for the family affected by HHCS. Squares indicate males, and circles, females; slashed symbols indicate deceased individuals. Double lines between symbols indicate consanguineous mating. Triangles indicate pregnancies that are not carried to term.  Proband is pointed with an arrow. Black-filled symbols indicate affected individuals by the presence of hyperferritinemia and cataracts; gray-filled symbols indicate individuals where only hyperferritinemia has been reported.

Further investigation of her family history revealed that the patient’s sister and her niece required bilateral cataract operations at the ages of 38 and 16, respectively (Figure 2). The patient's father was never assessed for hyperferritinemia or cataracts and passed away at the age of 62 due to a stroke. To the patient’s knowledge, her mother had a history of hyperferritinemia without cataracts, and the patient reported that her aunts and cousins on the maternal side have been suffering from bilateral cataracts and hyperferritinemia, have been treated with serial phlebotomies and followed up at hematology clinics of different centers for many years.

The patient was advised that phlebotomy was not the correct treatment for her condition because she did not have iron overload. Ferrous iron preparation was prescribed to accelerate her recovery from her current state of iron deficiency anemia, and she was referred to the ophthalmology clinic for planning her cataract surgery. An ophthalmology referral was also made for her three children for timely evaluation of possible early-onset bilateral cataracts.

## Discussion

Low ferritin levels reflect low total body iron stores. On the other hand, hyperferritinemia (women: >200 μg/L, men: >300 μg/L [5,6]) is not always associated with high total body iron stores. It is a positive acute phase reactant and it can increase without iron overload due to a variety of causes such as malignancy, infectious and inflammatory processes, or hereditary conditions [7]. Therefore, hyperferritinemia must be interpreted in conjunction with clinical history, physical exam, and other laboratory parameters (CRP, hemoglobin, serum iron, TIBC, transferrin saturation [TS], ALT, AST) for evaluation of an associated iron overload. When laboratory tests reveal hyperferritinemia with associated iron overload (high ferritin, low TIBC, high serum iron, high TS), and causes of secondary iron overload are excluded (alcohol use disorder, non-alcoholic fatty liver disease, hepatitis C virus), if the clinical suspicion remains high for HH, further genetic testing for *HFE* mutations can be pursued [4].

American College of Gastroenterology states in their HH 2019 clinical guidelines that patients with only *HFE* H63D or S65C mutations, and without C282Y mutation can present with elevated TS and serum ferritin levels, however, they are not found to be at an increased risk of iron overload, and they should be advised as such [4]. Even though our patient was found to be homozygous and her children were found to be heterozygous for the H63D mutation, their laboratory results did not only show hyperferritinemia, but also either normal iron indices or frank iron deficiency anemia. Therefore, their isolated hyperferritinemia could not be explained by having either one or two H63D mutated alleles, and hence, an alternative diagnosis was pursued. The co-occurrence of HH mutations, especially H63D, and HHCS mutations have been reported in the literature in the setting of hyperferritinemia without associated iron overload [6,8], which often results in patients receiving the diagnosis of HH, late or missed diagnosis of HHCS, and treatment with unnecessary medical interventions causing iatrogenic iron deficiency anemia.

The *FTL* gene resides on the long arm of chromosome 19, and codes for ferritin light chain protein (L-ferritin). Together with the ferritin heavy chain (H-ferritin), they make up the major intracellular iron storage protein, ferritin. HHCS has been shown to result from numerous point mutations, deletions, and insertions in the *FTL* mRNA’s 5’ untranslated hairpin loop region, where the iron-responsive element (IRE) resides [9,10]. Cytoplasmic proteins called iron regulatory proteins (IRPs) are responsive to cellular iron levels. IRPs regulate the amount of ferritin production, and hence iron storage, post-transcriptionally through interacting with IRE. When the level of cellular iron is low, IRPs bind to IRE, stabilize the IRE hairpin loop, prevent the ribosomal attachment to IRE, and downregulate the translation of L-ferritin mRNA. When there is abundant iron in the cell, IRPs detach from the IRE and allow for the ribosomal translation of L-ferritin mRNA into L-ferritin protein to facilitate the production of ferritin. In HHCS, mutations in the IRE of L-ferritin mRNA decrease the IRPs’ affinity to IRE, which in turn leads to decreased negative regulation of L-ferritin mRNA translation, the elevation of L-ferritin protein synthesis, and increased storage of iron despite low serum iron. Previously reported cases of HHCS and the present case to demonstrate that HHCS mutations are inherited in an autosomal dominant trait.

The CAGUGU sequence within the IRE is a non-coding sequence that is highly conserved, and this sequence was found to have an important role in IRP-IRE binding [9-12]. c.-160A>G mutation in this sequence was identified in 17 patients from six families from Kayseri and Nevşehir in Turkey and claimed to be the most prevalent mutation causing HHCS in Turkey [12]. Nevertheless, many mutations in different parts of the IRE of *FTL* have been identified, therefore, routine genetic testing is usually impractical and expensive to diagnose HHCS. Genetic testing is not necessary for diagnosis provided that isolated hyperferritinemia without iron overload and early onset cataracts are present in at least 2 or more members in a pedigree [6,13,14].

## Conclusions

In this report, we aimed to increase clinical awareness about HHCS, a rare differential diagnosis of hyperferritinemia without iron overload, to prevent unnecessary and even adverse medical interventions in HHCS patients. Because of the possibility of HH and HHCS mutations’ co-occurrence, physicians must maintain a high clinical suspicion of HHCS in patients with HH mutations, who have persistent hyperferritinemia and no iron overload despite repeated phlebotomies. Hyperferritinemia without iron overload with or without early-onset bilateral cataracts displaying an autosomal dominant inheritance pattern in three generations of this patient’s pedigree led to the diagnosis of HHCS. The history of early-onset cataracts in the patient's cousins and cataract surgery of the patient’s sister and niece at relatively young ages were not mentioned in her medical records. This case highlights the importance of taking a thorough family history in the context of early onset cataracts, isolated hyperferritinemia, and when any other rare clinical finding is detected in general. Family history is an important screening tool for construing aberrant clinical findings; it enables earlier diagnosis of rare hereditary conditions.

## References

[1] Craig JE, Clark JB, McLeod JL (2003). Hereditary hyperferritinemia-cataract syndrome: prevalence, lens morphology, spectrum of mutations, and clinical presentations. Arch Ophthalmol.

[2] Bonneau D, Winter-Fuseau I, Loiseau MN, Amati P, Berthier M, Oriot D, Beaumont C (1995). Bilateral cataract and high serum ferritin: a new dominant genetic disorder?. J Med Genet.

[3] Girelli D, Olivieri O, De Franceschi L, Corrocher R, Bergamaschi G, Cazzola M (1995). A linkage between hereditary hyperferritinaemia not related to iron overload and autosomal dominant congenital cataract. Br J Haematol.

[4] Kowdley KV, Brown KE, Ahn J, Sundaram V (2019). ACG clinical guideline: hereditary hemochromatosis. Am J Gastroenterol.

[5] Sandnes M, Ulvik RJ, Vorland M, Reikvam H (2021). Hyperferritinemia-a clinical overview. J Clin Med.

[6] Adams PC, Barton JC (2011). A diagnostic approach to hyperferritinemia with a non-elevated transferrin saturation. J Hepatol.

[7] Giansily-Blaizot M, Cunat S, Moulis G, Schved JF, Aguilar-Martinez P (2013). Homozygous mutation of the 5'UTR region of the L-ferritin gene in the hereditary hyperferritinemia cataract syndrome and its impact on the phenotype. Haematologica.

[8] Barton JC, Beutler E, Gelbart T (1998). Coinheritance of alleles associated with hemochromatosis and hereditary hyperferritinemia-cataract syndrome. Blood.

[9] Celma Nos F, Hernández G, Ferrer-Cortès X (2021). Hereditary hyperferritinemia cataract syndrome: ferritin L gene and physiopathology behind the disease-report of new cases. Int J Mol Sci.

[10] Girelli D, Corrocher R, Bisceglia L (1997). Hereditary hyperferritinemia-cataract syndrome caused by a 29-base pair deletion in the iron responsive element of ferritin L-subunit gene. Blood.

[11] Arnold J, Sangwaiya A, Manglam V, Thursz M, Beaumont C, Kannengiesser C, Busbridge M (2010). Hepcidin levels in hereditary hyperferritinemia: Insights into the iron-sensing mechanism in hepatocytes. World J Gastroenterol.

[12] Balta B, Erdoğan M, Kiraz A, Korkmaz S, Ağadayı A (2019). Frequent mutation in the FTL gene causing hyperferritinemia cataract syndrome in Turkish population is c.-160A>g. Turk J Haematol.

[13] Worth HA, Marlette Z, Aljadir D, Lands R (2020). Hereditary hyperferritinemia-cataract syndrome in 3 generations of a family in East Tennessee. Case Rep Hematol.

[14] Hetet G, Devaux I, Soufir N, Grandchamp B, Beaumont C (2003). Molecular analyses of patients with hyperferritinemia and normal serum iron values reveal both L ferritin IRE and 3 new ferroportin (slc11A3) mutations. Blood.

